# Factors affecting the micronutrient status of adolescent girls living in complex agro-aquatic ecological zones of Bangladesh

**DOI:** 10.1038/s41598-023-33636-8

**Published:** 2023-04-24

**Authors:** Gulshan Ara, David C. Little, Abdullah-Al Mamun, Baukje de Roos, Eleanor Grieve, Mansura Khanam, S. M. Tafsir Hasan, Santhia Ireen, Samira Dilruba Ali, Anika Bushra Boitchi, Marjoleine A. Dijkhuizen, Tahmeed Ahmed, Nanna Roos

**Affiliations:** 1 Nutrition and Clinical Services Division, icddr, b, 68 Shaheed Tajuddin Ahmed Sarani, Mohakhali, Dhaka 1212 Bangladesh; 2 Institute of Aquaculture, University of Stirling, Stirling, UK; 3 Department of Fisheries and Marine Science, Noakhali Science and Technology University, Noakhali, Bangladesh; 4 The Rowett Institute, University of Aberdeen, Aberdeen, UK; 5 HEHTA, University of Glasgow, Glasgow, UK; 6 FHI 360 Mohakhali, Bangladesh; 7 Department of Nutrition, Exercise, and Sports, University of Copenhagen, Copenhagen, Denmark

**Keywords:** Nutrition, Public health

## Abstract

Inadequate intake of both macro and micronutrients is the major determinant of micronutrient deficiencies in adolescent girls. This study assessed multiple micronutrient status including vitamin D, iron, vitamin A, and urinary iodine concentration among adolescent girls through two seasonal cross-sectional surveys conducted during dry and wet seasons. Mixed-effects linear and logistic regression analysis were conducted to assess associations between micronutrient status, salinity and seasonality. The mean age of the girls was 14 years. Vitamin (OH)D insufficiency was significantly higher in freshwater areas in wet season compared to dry season (wet season: 58% and dry season: 30%, *P* < 0.001). In wet season, risk of vitamin (OH)D insufficiency was three times higher compared to dry season (AOR: 3.03, 95% CI 1.71, 5.37, *P* < 0.001). The odds of vitamin (OH)D insufficiency was 11 times higher in fresh water areas compared to high saline areas (AOR: 11.51, 95% CI 3.40, 38.93, *P* < 0.001). The girls had higher risk of iron deficiency in wet season. Despite the environment being enriched with micronutrient-contained aquatic food, adolescent girls in coastal areas experience different micronutrient deficiencies. The high prevalence of vitamin (OH)D insufficiency in freshwater locations and seasonal iron deficiency in high saline areas needs attention.

## Introduction

Adolescence is a decisive life stage for achieving optimal health and nutrition^1^. After early childhood, adolescence is the most critical period for growth and changes in body composition, physiology, and the endocrine system^2^. The majority of rural Bangladeshi women have their first child before reaching the age of 18 years, which frequently has a detrimental effect on both maternal and child health^3^. According to the Bangladesh Demographic Health Survey (BDHS) 2017–18, 24% of ever-married women aged 15 to 19 years old were chronically energy deficient [body mass index (BMI) < 18.5] and 9% were moderate to severely thin (BMI < 17.0)^4^. Hence, to break the vicious cycle of intergenerational malnutrition, adolescence is the last opportunity to catch up on any growth faltering experienced during childhood and support the growth spurt alongside skeletal development observed during this period^2^. Micronutrient deficiency is a major public health concern worldwide, with adolescents being one of the most susceptible groups^5^, primarily due to poor micronutrient content and bioavailability in diets, as well as poor hygiene and infections^6,7^. A household survey in Bangladesh showed that the median intakes of key micronutrients by pregnant adolescent girls were below the estimated average requirements (EARs)^8^. The requirement of micronutrients increase among adolescent girls during periods of peak physical growth when the body needs an increased input of dietary iron, calcium, zinc, iodine, vitamin A, and vitamin D^9^.

Iodine deficiency among adolescent girls can lead to goiter, mental and physical growth retardation which later on may extend upon their fetuses and newborns via intergenerational effect^10,11^. Adolescent girls are particularly at risk in Bangladesh because 40% of the school children had mean UIC below 100 µg/l^12^. Vitamin D is important for calcium homeostasis to maintain skeletal integrity. A study in Bangladesh found an aging-related rise in both the prevalence of vitamin D insufficiency (S-25OHD 25–75 nmol/L) and the deficiency (S-25OHD < 25 nmol/L) among adolescents, with nearly all adolescents aged 12–16 years being either deficient (46%) or insufficient (52%)^13^. Both iron deficiency (ID) and iron deficiency anaemia (IDA) are global health problems. However, the adolescent girls^14^ are more likely to suffer from growth retardation^15^. According to a study, 32% of the adolescent girls living in peri-urban areas had iron deficiency anaemia^16^. Another essential micronutrient is vitamin A, which has a variety of physiological functions. Vitamin A deficiency (VAD) results in night blindness, severe anaemia, wasting, reproductive infirmity, infectious morbidity, and increased risk of mortality^17^. In Bangladesh, one-third of the non-pregnant and non-lactating populations are experiencing mild grade of vitamin A deficiency^18^. Hence, consumption of micronutrient-rich foods should suffice all micronutrient requirements.

Fish is significantly a good source of dietary protein and micronutrients in low- and middle-income countries^19–23^. In Bangladesh, fish is consumed frequently, often daily or several times per week in peak seasons of high availability, although the portions consumed can be too small to provide a sufficient amount of key micronutrients^24^. The southwest coastal region of Bangladesh is considered one of the most diversified hubs of aquatic resources from fisheries and aquaculture^25^. In addition, the dynamics of the ecosystems in these coastal zones are complex, ranging from saline to freshwater aquatic environments, with seasonal fluctuations in the intrusion of oceanic water meeting riverine freshwater^26,27^. The availability of aquatic foods is highly diverse across the ecological zones, and it fluctuates between dry and wet seasons. Yet, there is no information available on how the delicate eco-system affects the seasonal nutrition and health status of the vulnerable people. We postulated that the overall micronutrient status of these girls may be impacted by salinity, seasonality, and consumption of various sea foods.

We hypothesized that salinity, seasonality and intake of different sea food may have an impact on the overall the micronutrient status of these girls. Therefore, we undertook the study to assess the micronutrient status including urinary iodine concentration, total vitamin (OH)D, serum ferritin, and serum retinol. We also assessed food intake in adolescent girls living in aquatic farming communities across a gradient from coastal to inland environments, representing different ecosystems.

## Methods

### Sampling and population

**Figure 1:**
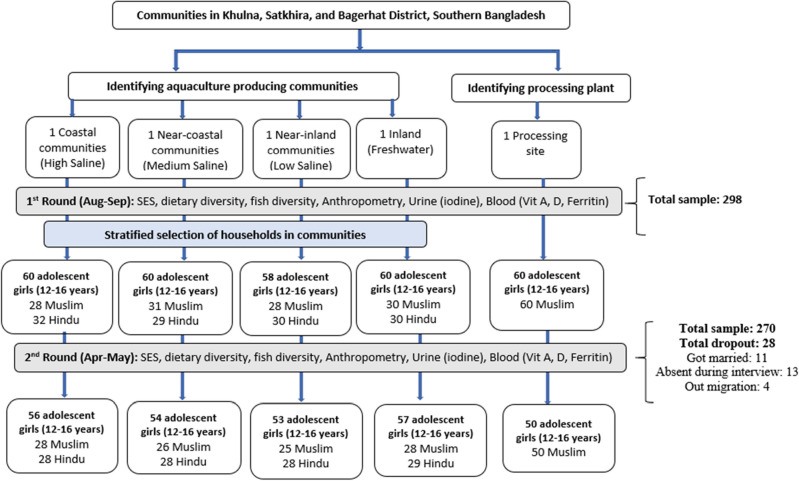
Schematic diagram of stratified sampling and the two seasonal survey rounds.

Two stratified cross-sectional surveys were carried out from August—September 2017 (dry season) and April—May 2018 (wet season). The wet season is associated with peak shrimp production, and the dry season with peak fish and prawn production^28^. The study population was adolescent girls aged 12–16 years old. Based on the dry season surface water salinity of adjacent rivers and canals, seafood production of the southwest coastal floodplain is divided into four major agro-ecological areas. Agro-ecologies that characterize the prevailing salinity gradient of the region were used to identify household clusters engaged in farmed seafood value chains; they included high-, medium-, and low-salinity, freshwater, and an urban location with a concentration of shrimp processing facilities^29^. The study focused on the two most important nodes of the seafood value chain in Bangladesh: the farming community and the processing zone. They are: (1) high saline area (HS); salinity is > 10 ppt (2) medium saline area (MS), salinity ranging > 5 to 10 ppt (3) low saline area (LS) typically the salinity remains 0.5–5 ppt (4) freshwater area (FW); salinity < 0.5 ppt^28^. Data collection covered a seafood processing plant (PP), a location where companies received cultured seafood products from the fish farms for processing and packaging for export markets. Sampling was stratified by the five settings (four aqua-agroecological zones and one processing plant community), with 60 adolescent girls recruited in each site. Additionally, the sample was stratified for religion aiming for equal numbers of Hindus and Muslims in each area. Although at the national level, Hindu communities represent less than 10% of the population, the proportion of Hindus in the study areas was high. Sub-districts from each aqua-agroecology were purposively selected and local demographics were assessed after engagement with sub-district (Upazila) level officials. Key informant (KI) interviews led to identification of unmarried adolescent girls at household levels of selected communities. Communities with two adjacent Para (small unit of a village), one Muslim-dominated and the other Hindu-dominated, were selected for each zone, considering the size of the community to ensure an adequate sample size. The PP area was predominantly Muslim (> 98%) and this community was therefore not stratified by religion. Households with at least one unmarried adolescent girl were identified and invited to participate in this study. The same girls were surveyed repeatedly, during the wet and dry seasons, for a total sample size of 300 adolescent girls in each season. In total, 298 teenage girls took part in the first round of data collection. During the second survey, however, the field team was unable to reach every girl who participated in the first survey. A total of 270 girls were surveyed, with a dropout rate of 9% due to marriage, absences during data collection and out migration Fig. 1).

### Ethical approval and consent to participate

This study was conducted according to the guidelines laid down in the Declaration of Helsinki. Here, all of the study procedures involving the adolescent girls were ethically approved by the Ethical Review Committee of the icddr,b and NHS, Invasive or Clinical Research (NICR) Committee, University of Stirling, UK (PR-17037). Written informed consent was obtained from all participants and their parents or guardian before their participation in this study.

### Data, blood and urine sample collection

A team of field research staff with relevant experience recruited by Noakhali Science and Technology University (NSTU) was responsible for the collection of field data on socioeconomic indicators, dietary intake, fish consumption, and anthropometric parameters using a structured and semi-structured questionnaire. The field supervisors and data collection teams were trained on the interview techniques, questionnaires, and nutritional status measurements. The data collection tools were pre-tested among the volunteer population, a total of 12 interviews were conducted. To maintain the standard of data collection, they adhered to anthropometry guidelines and a standard operating procedure (SOP). In addition to training, the data collectors completed a validation exercise. Blood and urine samples were collected by trained medical technologists from International Centre for Diarrhoeal Disease and Research (icddr,b).

Socio-demographic information included the level of education, occupation of the household head, number of family members, ownership of the house, number of dwelling rooms, household construction materials, toilet facilities, sources of drinking water, land ownership, monthly household expenditure, and household assets was collected as indicators of socioeconomic status (SES). The dietary diversity of adolescent girls was assessed using a 24-h dietary recall approach, and the types of fishes consumed were assessed using a 7-day semi-quantitative Food Frequency Questionnaire (FFQ), which had been previously designed and validated^30^. The amount of food consumed by the adolescent girls was calculated using pictures of the serving and the weight (in grams). The weight of the raw food was computed using the proper conversion factors^12^. According to accepted methods^31^, all food items were divided into ten major food groups. They are: (i) cereals (rice, wheat, other cereals), (ii) vegetables (plants, vegetables, leafy vegetables), (iii) pulses (pulses, legumes, and nuts), (iv) eggs, (v) flesh food (chicken, beef, other organ meat, dried fish), (vii) milk (milk and milk products), (viii) beverages, (ix) fruits rich in vitamin A, and (x) other foods. The respondents were asked to recall which kinds of fish they had eaten regularly in the previous five days, three months, and one year. Fish intake (sea fish, finfish, and shellfish) was also ranked at the household level by the types of fishes most frequently eaten. A wealth index was created using principal component analysis (PCA) of household assets information. A weight was attached to each item from the first principal component. The households were classified into SES quintiles based on the wealth index, from quintile 1 (poorest) to 5 (richest)^32^.

The adolescent girls were weighed using electronic scales (Tanita Inc. Tokyo, Japan) with a precision of 100 g. Height was measured using locally made standardized wooden length/height boards with a precision of 0.1 cm (1 mm). In both survey points, blood and urine samples were collected in a health center, developed as temporary sample collection centres on the same day. 3.5 ml of venous blood were collected in a Venoject tube to obtain 1200 µl serum. After the blood collection from all participants, the blood tubes were placed in a cool box and allowed to clot. At the end of each day, the whole blood was centrifuged and the serum was aliquoted into at least three cryovials by pipetting using a disposable pipette. A barcoded label was provided for each of the study participant’s questionnaire form and each of the aliquoted cryovials. The serum was stored in a freezer (-20ºC or colder) as soon as possible. During the transportation of the serum to the Nutritional Biochemistry Laboratory at icddr,b, the cold chain was maintained. Samples were stored in a -70 °C freezer and analyzed in the Nutritional Biochemistry Laboratory to estimate blood parameters. The participants provided urine samples in single-use plastic cups at the survey spot. The samples were transferred to wide-mouthed screw-capped plastic bottles that had been previously washed with de-ionized water and dried.

### Measurement of urine iodine, serum retinol, serum ferritin, CRP, AGP, and total serum vitamin (OH)D

Urinary iodine was determined by a colorimetric method^33^ at the icddr,b. A mean and/or median urinary iodine concentration (UIC) (µg/l) can be used to report the iodine status^34,35^. Urinary iodine concentration (UIC) was defined as a concentration level < 100 µgm/L^12,34^. Total vitamin (OH)D level was measured by electrochemiluminescence binding assay using a Roche Cobas e601 automated immune analyzer^36^. The assay employs a polyclonal antibody directed against 25-OH vitamin D^37^. Vitamin (OH)D insufficiency was defined as a serum level < 50 nmol/L and < 30 nmol/L for deficiency^38^. Serum ferritin, C-reactive protein (CRP) and alpha-1-acid glycoprotein (AGP) were analyzed by a sandwich ELISA technique^39^. Iron deficiency (ID) was defined as a median serum level < 15 ug/L, after adjusting for inflammatory markers CRP and AGP^40^. Serum retinol was determined using high-performance liquid chromatography (HPLC)^41^. The threshold for vitamin A deficiency (VAD) was a normal status: serum retinol ≥ 1·05 µmol/l (30 ug/dl), mild deficiency: serum retinol ≥ 0·7– < 1·05 µmol/l (≥ 20- < 30 ug/dl)^42^.

### Quality control of field data collection

The quality control team of investigators monitored the performance of the field personnel and supervisors through regular observations and regular checking of the data for completeness. The field supervisors independently repeated the data collection on socio-demographic information and anthropometry in a random selection of 5% of the study participants. Identical forms, equipment, definitions, and methods were used throughout the study period.

### Sample size calculation

The following formula for proportion in a cross-sectional survey was used to calculate the sample size: n = Zα^2^ P (1-P)/d^2^ (precision) × design effect. Here, Zα = 1.96 considering 95% confidence interval, *P* = 0.27 (27% prevalence of vitamin D^38^ and 37% urinary iodine deficiency^43^), d = 0.05 (5% level of significance) and design effect = 1.2. The total sample size came to 295 adolescent girls per survey. For ease of sample distribution across different study areas, a total of 300 adolescent girls was included in the study.

### Data analysis

Data analysis was performed using STATA 13.0 SE (Stata Corp, College Station, TX, USA). Pearson’s *χ*^2^ test was used to compare the proportions of background and key outcome variables. We used a multivariable mixed-effect linear regression model with robust standard error to assess the association between the aquatic ecological zones and seasonality with the continuous measures for the status of the four micronutrients defined by the biochemical indicators as continuous variables (primary analysis). In addition, we used mixed-effects logistic regression to investigate factors associated with micronutrient deficiency defined by cut-off values for each of the micronutrients assessed (secondary analysis). We specified a random effect at the individual level to account for within-individual correlations as data on the outcomes were collected on two time points. The “melogit” command in Stata was used to enable the inclusion of categorical covariates in the multivariable models. The regression was conducted using the stepwise backward method to determine the factors significantly associated with the occurrence of micronutrient deficiency. Each analysis (primary and secondary) was adjusted for age, dietary diversity quintiles, BMI, and wealth index. The data were reported by the regression coefficient and adjusted odds ratio (AOR) and 95% confidence intervals. Initial regression analyses included the interacting predictors and the independent variables that were supposed to interact as an interaction term. Model assumptions for each analysis were checked using residual and normal probability graphs. Non-normally distributed variables were logarithmically transformed before analysis. Data for urinary iodine, total vitamin (OH)D, ferritin and retinol were log-transformed prior to analysis.

## Results

Table 1 shows the background characteristics of the adolescent girls by different salinity areas assessed during the first round of the dry season (August to September 2017) of the survey. The average age of the girls was similar across all sites, except for the girls in the PP area who were slightly younger. The majority of the girls had seven years of schooling on average. Almost all of them had access to mobile phones. The wealth distribution appeared slightly different between the salinity zones, with more girls in the richest quintile in the FW and PP zones. The median urinary iodine concentration, serum retinol and serum ferritin level were normal for this population of adolescent girls. All girls had adequate levels of total serum vitamin (OH)D when the median serum level < 50 nmol/L for insufficiency was considered.

**Table 1:** Background characteristics and nutritional status of adolescent girls by the salinity areas at the first survey round (dry season).

Categories	High saline (n = 60)	Medium Saline (n = 60)	Low saline (n = 58)	Fresh water (n = 60)	Processing Plant (n = 60)
*Background characteristic*					
Age (mean)	14.0 ± 1.5	13.9 ± 1.4	14.2 ± 1.5	14.2 ± 1.6	13.3 ± 1.2
Religion					
Muslim	28 (46.7)	31 (51.7)	28 (48.3)	30 (50.0)	60 (100.0)
Hindu	32 (53.3)	29 (48.3)	30 (51.7)	30 (50.0)	0 (0.0)
Years of schooling (mean)	7.2 ± 1.7	7.1 ± 1.8	7.3 ± 2.0	7.8 ± 1.8	6.2 ± 2.0
Family size [mean (min–max range)]	5.2 (3–12)	5.0 (3–9)	4.5 (2–10)	4.9 (3–8)	4.6 (2–8)
*Socio-economic conditions*					
Access to:					
Electricity (n)	45 (75.0)	48 (80.0)	51 (87.9)	58 (96.7)	59 (98.3)
Mobile phone (n)	59 (98.3)	59 (98.3)	55 (94.8)	56 (93.3)	57 (95.0)
Drinking water (n)	36 (60.0)	29 (48.3)	58 (100.0)	56 (93.3)	60 (100.0)
Improved toilet (n)	54 (90.0)	53 (88.3)	55 (94.8)	59 (98.3)	58 (96.7)
Wealth quintiles^§^					
1st quintile (poorest)	15 (27.3)	15 (29.4)	13 (24.1)	3 (5.0)	5 (15.6)
2nd quintile (poorer)	6 (10.9)	13 (25.5)	10 (18.5)	13 (21.7)	8 (25.0)
3rd quintile (middle)	12 (21.8)	5 (9.8)	12 (22.2)	19 (31.7)	3 (9.4)
4th quintile (richer)	14 (25.5)	10 (19.6)	8 (14.8)	10 (16.7)	8 (25.0)
5th quintile (richest)	8 (14.6)	8 (15.7)	11 (20.4)	15 (25.0)	8 (25.0)
*Nutritional status of adolescent girls*					
Weight in kg (mean ± SD)	41.0 ± 6.8	40.4 ± 6.7	45.4 ± 9.3	43.8 ± 7.4	40.3 ± 8.4
Height in cm (mean ± SD)	149.7 ± 5.8	149.4 ± 5.6	150.7 ± 6.8	151.7 ± 7.2	147.4 ± 8.0
BMI for age z-score (mean ± SD)	− 0.65 ± 1.16	− 0.70 ± 1.16	− 0.05 ± 1.24	− 0.35 ± 0.98	− 0.44 ± 1.33
Urinary iodine (µg/L) [median, interquartile range]	338.2 (191.5, 472.6)	200.1 (115.7, 301.6)	221.7 (117.7, 411.5)	438.7 (294.6, 756.2)	332.1 (203.5, 614.5)
Vitamin (OH) D (nmol/L) [median, interquartile range]	60.7 (55.7, 71.6)	73.6 (62.8, 87.8)	62.2 (49.7, 71.1)	50.3 (40.5, 61.1)	56.1 (47.7, 65.2)
Ferritin (mmol/L) [median, interquartile range	49.8 (21.6, 71.2)	43.1 (21.2, 63.3)	44.1 (25.1, 70.8)	41.7 (29.0, 68.2)	50.2 (36.8, 73.9)
Serum retinol (µg/dL) [median, interquartile range]	41.3 (35.5, 47.5)	41.9 (36.7, 48.2)	44.4 (38.0, 50.4)	40.0 (34.5, 44.5)	39.7 (33.7, 42.2)

*BMI* Body Mass Index, *SD* Standard Deviation.

^§^Wealth quintile defined by the entire sample (n = 298).

**Figure 2:**
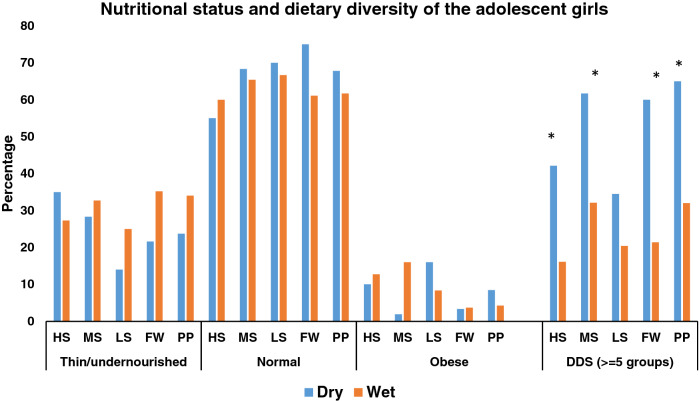
Nutritional status of adolescent girls by the salinity and seasonality. BMI-for-age of adolescent girls was measured during dry (August to September 2017) and wet (April to May 2018) season. Ref: Underweight (< -2SD >), Normal weight (-2 SD to + 1 SD), and Overweight (> + 1 SD). Dietary diversity score ≥ 5 (in last 24 h) among adolescent girls. Statistical significance (*P* < 0.005) has been denoted by asterisk: *Pearson’s chi-square test. Note: Different agro-aquatic ecological zone- HS, High saline; MS, Medium saline; LS, Low saline; FW, Fresh water, and PP, Processing plant.

**Figure 3:**
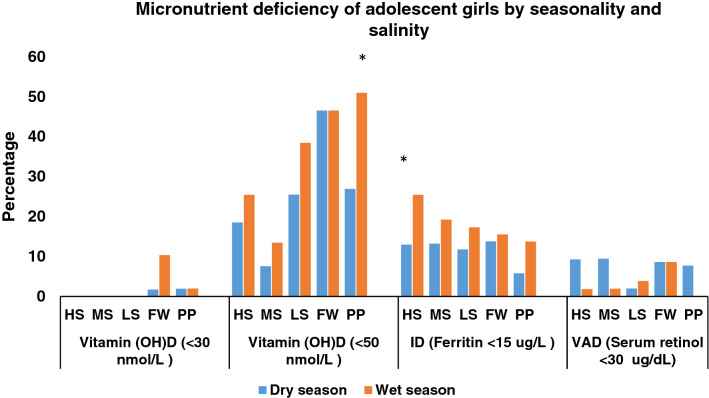
Micronutrient deficiency of adolescent girls by seasonality and salinity. Vitamin (OH) D deficiency (< 30 nmol/L), vitamin (OH) D insufficiency (< 50 nmol/L), Iron Deficiency (Ferritin < 15 ug/L) and VAD (Serum retinol < 30 ug/dL). Statistical significance (*P* < 0.005) has been denoted by asterisk: *Pearson’s chi-square test. Note: Different agro-aquatic ecological zone- HS, High saline; MS, Medium saline; LS, Low saline; FW, Fresh water, and PP, Processing plant.

**Figure 4:**
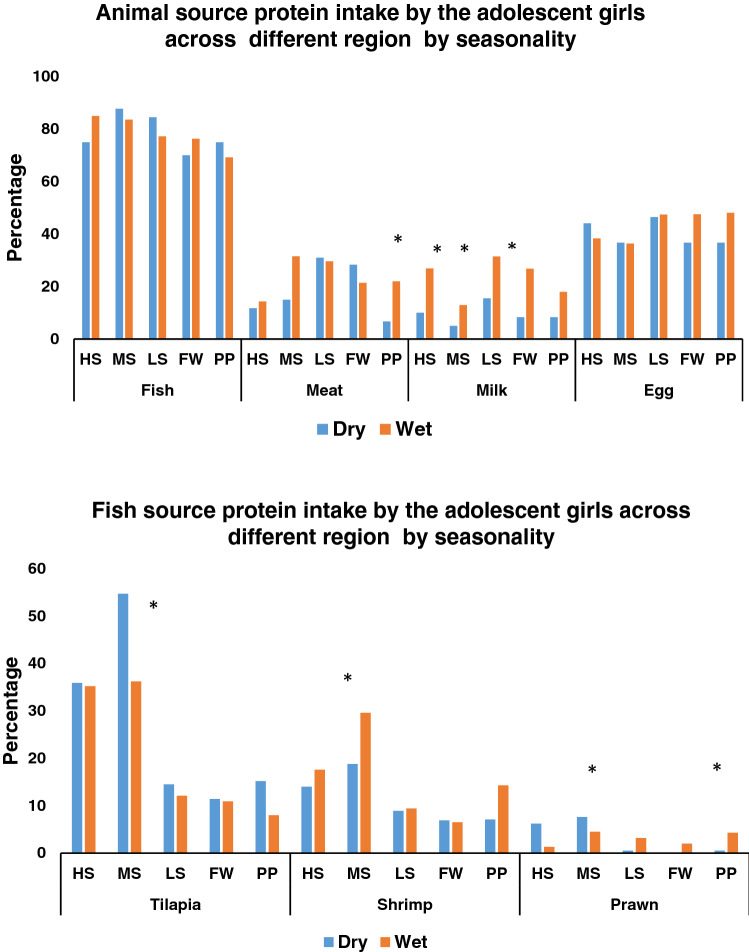
(**A**) Percentage of animal source food consumption, (**B**) Percentage of major types of fish consumed (in last 7 days). Statistical significance (*P* < 0.005) has been denoted by asterisk. * Pearson’s chi-square test. Note: Different agro-aquatic ecological zone- HS, High saline; MS, Medium saline; LS, Low saline; FW, Fresh water, and PP, Processing plant.

In all areas, with the exception of the HS area, the proportion of thinness (BMI-for-age < 2 SD) among adolescent girls was higher during the wet season, although the difference was not statistically significant. The diversity of the diet varied significantly with the season; diets were more likely to contain food from more than five food categories during the wet season than the dry season (Fig. 2). According to area-based analyses of the different micronutrient deficiencies of adolescent girls for each season, deficiencies of micronutrients, particularly vitamin (OH)D insufficiency, were common in all seasons and across salinity zones. In the PP area, vitamin (OH)D insufficiency was present in 56% of girls during the wet season compared to 30% of girls during the dry season and this difference was significant (*P* < 0.005). Hence, compared to the dry season, the wet season tends to have a somewhat higher prevalence of vitamin (OH)D insufficiency. According to the vitamin (OH)D deficiency criteria [vitamin (OH)D < 30 nmol/L], 10% of the girls in the FW area had deficiencies during the wet season, but only 2% of the girls from the FW and PP areas had deficiencies during the dry season. Similar to vitamin D insufficiency, a rising tendency of ID was seen in the wet season as opposed to the dry season. Thirteen percent of the girls had ID in dry season and overall, one-fourth of girls from the HS areas were iron deficient during wet season. In the dry season, VAD (serum retinol < 30 ug/dL) affected 9% of girls in HS, MS, LS, and PP areas, but the prevalence of VAD was reduced in HS MS and LS areas in wet season. Nevertheless, in the FW area, it remained unchanged (Fig. 3). Compared to meat, milk, and eggs, fish was the most popular animal protein consumed by adolescent females during two different seasons and in all agro-ecological zones. In both seasons more than eighty percent of the girls consumed fish in last 24 h. A significantly higher proportion of girls in all areas consumed milk in the wet season than the dry season. Tilapia was found to be the most commonly consumed fish, among adolescent girls in both seasons across all the salinity areas. The proportion of girls who ate tilapia over the previous week in medium saline and processing plant locations was significantly higher in the dry season than the wet season (MS-dry season 55% and MS-wet season 36%, *P* < 0.005; PP-dry season 15% and PP-wet season 8%, *P* < 0.005). Again, in these two regions, considerably more girls consumed prawn during the wet season compared to the dry season (MS-dry season 19% and MS-wet season 30%, *P* < 0.005; PP-dry season 7% and PP-wet season 14%, *P* < 0.005) (Fig. 4A-4B).

Table 2 presents the estimated geometric mean ratio (GMR) or Exp (Coef.) of the micronutrient indicators and the results of the adjusted mixed-effect linear regression model. The urinary iodine level was higher in the wet season compared to the dry season (*P* = 0.007). Girls from the MS and LS areas had significantly lower UIC than those from HS areas (MS, Coef: 0.57, 95% CI 0.57, 0.90; *P* = 0.004 and LS, Coef: 0.61, 95% CI 0.47, 0.80; *P* < 0.001). On the other hand, girls who lived in the FW and PP zones had considerably higher levels of UIC. The total serum vitamin (OH)D level was lower during the wet season compared to the dry season (Coef: 0.89, 95% CI 0.86, 0.93; *P* < 0.001). Vitamin (OH)D level was significantly higher among the girls living in MS area and lower in LS, FW and PP areas compared to girls from HS areas. When compared to the dry season, the serum ferritin was significantly lower in the wet season (Coef: 0.79, 95% CI 0.71, 0.88; *P* < 0.001). Girls from PP areas were shown to have better ferritin status than those from HS areas. Even though there was a trend toward increased serum level concentration of vitamin (OH)D and ferritin during the dry season, mean serum retinol levels were noticeably higher than during the wet season. When compared to HS locations, the serum retinol level was significantly higher in MS areas.

**Table 2:** Factors affecting micronutrient status in adolescent girls.

		Urinary iodine concentration			Vitamin (OH) D concentration			Ferritin concentration			Retinol concentration		
		Adjusted*			Adjusted*			Adjusted*			Adjusted*		
		Exp (β)	95% CI	*P*-value	Exp (β)	95% CI	*P*-value	Exp (β)	95% CI	*P*-value	Exp (β)	95% CI	*P*-value
Seasonality													
Dry		Ref			Ref			Ref			Ref		
Wet		1.19	(1.05, 1.35)	0.007	0.89	(0.86, 0.93)	< 0.001	0.79	(0.71, 0.88)	< 0.001	1.11	(1.07, 1.15)	< 0.001
Salinity													
HS		Ref			Ref			Ref			Ref		
MS		0.71	(0.57, 0.90)	0.004	1.18	(1.08, 1.29)	< 0.001	1.07	(0.77, 1.49)	0.674	1.04	(0.97, 1.11)	0.268
LS		0.61	(0.47, 0.80)	< 0.001	0.93	(0.85, 1.00)	0.050	1.16	(0.85, 1.60)	0.348	1.09	(1.01, 1.18)	0.030
FW		2.04	(1.63, 2.55)	< 0.001	0.78	(0.72, 0.84)	< 0.001	1.13	(0.83, 1.53)	0.445	0.95	(0.90, 1.01)	0.102
PP		1.52	(1.21, 1.91)	< 0.001	0.82	(0.78, 0.89)	< 0.001	1.39	(1.04, 1.85)	0.025	0.95	(0.89, 1.01)	0.074

*CI* Confidence Interval, *Ref*. Reference.

Mixed-effects linear regression analysis adjusted for each other independent variables and other variables (age, BMI, dietary diversity score, wealth quintiles). All biomarkers (urinary iodine, vitamin D, serum retinol and ferritin) were log-transformed; the co-efficient is expressed as the Exp (β) / Geometric Mean Ration (GMR) with 95% CIs. Statistical significance has been considered for 5% level of significance.

Table 3 demonstrates the risk factors identified by logistic regression for the micronutrient deficiencies. Iodine status of adolescent girls was unaffected by the season; their odds of having urinary iodine deficiency were 5 times higher in the LS than in the HS (AOR: 5.15, 95% CI 1.73, 15.35, *P* = 0.003) areas. We found a significant seasonal effect on the likelihood of vitamin (OH)D insufficiency among the adolescent girls. The odds of vitamin (OH)D insufficiency was 3 times higher (AOR: 3.03, 95% CI (1.71, 5.37), *P* < 0.001) in the wet season compared to the dry season. Girls who resided in FW regions had a five-fold increased risk of vitamin (OH)D deficiency compared to girls who lived in the HS areas (AOR: 4.9, 95% CI 2.3, 10.2; *P* < 0.001). The girls living in the FW and PP areas also had a higher risk of vitamin (OH)D insufficiency compared to the girls from HS area (FW, AOR: 11.51, 95% CI 3.40, 38.92, *P* < 0.001 and PP, AOR:5.72, 95% CI 1.85, 17.67, *P* = 0.002). The seasonal effect was also observed in the serum ferritin concentration of the girls; compared to the dry season, the risk of iron deficiency was around 4 times higher in the wet season (AOR: 3.78, 95% CI 1.01, 14.10; *P* = 0.048). However, salinity level was not associated with risk ID. In comparison to the dry season, girls had a lower risk of vitamin A deficiency during the wet season (AOR: 0.47, 95% CI 0.14, 0.94; *P* = 0.038).

**Table 3:** Risk factors for urinary iodine, serum vitamin (OH)D, iron and vitamin A deficiencies among the adolescent girls.

Categories	Urinary iodine deficiency^a^		Vitamin (OH)D insufficiency^a^		Iron deficiency^a^		Vitamin- A deficiency^a^	
	AOR (95% CI)	*P*-value	AOR (95% CI)	*P*-value	AOR (95% CI)	*P*-value	AOR (95% CI)	*P*-value
*Seasonality*								
Dry	Ref		Ref		Ref		Ref	
Wet	0.69 (0.37, 1.31)	0.261	3.03 (1.71, 5.37)	< 0.001	3.78 (1.01, 14.10)	0.048	0.37 (0.14, 0.94)	0.038
*Salinity area*								
HS	Ref		Ref		Ref		Ref	
MS	1.93 (0.59, 6.31)	0.277	0.26 (0.06, 1.13)	0.073	0.59 (0.12, 2.86)	0.516	0.89 (0.24, 3.30)	0.865
LS	5.15 (1.73, 15.35)	0.003	2.59 (0.74, 9.05)	0.136	0.61 (0.11, 3.23)	0.559	0.33 (0.05, 2.13)	0.244
FW	0.37 (0.09, 1.58)	0.178	11.51 (3.40, 38.93)	< 0.001	0.74 (0.17, 3.24)	0.691	1.67 (0.48, 5.79)	0.413
PP	1.25 (0.40, 3.87)	0.700	5.72 (1.85, 17.67)	0.002	0.24 (0.04, 1.45)	0.122	0.71 (0.15, 3.26)	0.656

*AOR* adjusted odds ratio, *pp* percentage point, *CI* Confidence Interval, *Ref*. Reference.

^a^Adjusted for each other and other variables (age, BMI, dietary diversity score, and wealth quintiles). Adjusted odds ratios were estimated using mixed effect logistic regression where independent variables were the seasonality and agro-aquatic region.

## Discussion

The study assessed micronutrient status and risks of micronutrient deficiencies among adolescent females residing in the coastal regions of Bangladesh. We found multiple micronutrient insufficiencies and deficiencies, which varied over a gradient from coastal to inland regions and with the seasons. One fourth of girls had ID in the wet season and 10% of girls had vitamin A deficiency in the dry season. In particular, the high prevalence of vitamin (OH)D insufficiency among adolescent girls living in processing plant areas during the wet season is of concern.

In Bangladesh, vitamin (OH)D deficiency is a public health concern^13^. In our study, we observed a higher prevalence of girls having vitamin (OH)D insufficiency especially in the freshwater zone across both seasons. More than half of the girls in the FW and PP areas had vitamin (OH)D insufficiency in the wet season. A study conducted in Iran reported women had significantly lower serum vitamin (OH)D concentrations than men in both summer and autumn (*P* = 0.021 and 0.016 respectively)^44^. Seasonal variability of vitamin D concentrations is also observed among the Polish children^45^. African-American girls had higher mean vitamin (OH)D levels in the spring/summer months than in fall/winter months^46^. Therefore, culture, religion, variation in sun exposure, clothing choice, skin pigmentation and age may be important contributors to total vitamin (OH)D level^47^. We observed a trend towards an increase in vitamin (OH)D insufficiency as the salinity of the area reduced. One plausible explanation could be the distinct dietary habits of the girls in the HS and MS areas, who had a higher intake of tilapia and substantially obtained the high levels of Vitamin D3. A study showed that farmed tilapia is a valuable source of vitamin D3; a study of the Polish population revealed lean tilapia has a high vitamin D content (38.0 ± 7.7 mg/100 g)^48^.

In Bangladesh, the school-age children aged 6 to 11 and 12 to 14 years had an iron deficit of 3.9% and 9.5%, respectively^12^. Half of the cases of anemia are accountable to iron deficiency, but the proportion varies among population groups and in different areas, according to the local conditions^49–51^. However, the prevalence of ID and IDA in the Bangladeshi population appeared to be substantially lower than the widely held assumption, perhaps linked to the bioavailable iron found in groundwater^12^. Remarkably, the iron status of the adolescent girls in this study differed with the season, with the highest prevalence of ID observed in the wet season in the three saline regions. A Brazilian study showed the mean serum ferritin concentrations of riverine populations were higher in the dry season than in the rainy season^52^. One reason contributing to the increasing levels of ID during the wet season may be the mixing of surface water and groundwater during the rainy season, which lowers the overall iron content of the mixed water^53^.

Twenty-one percent of the Bangladeshi school-going children are sub-clinically vitamin A deficient. In our study we found a low prevalence of VAD with seasonal fluctuation, girls living in the LS area had higher mean serum retinol levels (9%) compared to those in the HS area. Severe grade of vitamin A deficiency (serum retinol < 0·35 µmol/l) was not observed. Mandatory fortification of edible oil^54^, and high consumption of fish by adolescent girls might have contributed to the adequate vitamin A status of these girls^21,55^. According to a study by Sabuktagin et al., eating animal source foods was associated with higher retinol levels in school children^18^.

Iodine Deficiency Disorder (IDD) is a significant global public health issue that affects all age groups, adolescent girls living in iodine-deficient areas had poorer school performance, lower IQs, learning disabilities and were more exposed to the consequences of IDD for their offspring^56^. We found that none of the adolescent girls had urinary iodine concentration < 100 µg/L as the median level of urinary iodine concentration was higher than the population median level (> 100 µg/L) which indicates the girls are getting an adequate amount of iodine through salt, seafood and environment. The local diet and seafood may contain higher iodine concentrations in the HS areas possibly combined with a frequent presence of fish and seafood in the diet^57^. A study in China showed that the mean urinary iodine concentrations were significantly higher among children in coastal areas compared with children in inland areas in all age groups (*P* < 0.05)^57^. Another study reported a significant positive correlation between the iodine concentrations of salt samples and the urinary iodine concentrations of Bangladeshi adolescent girls and pregnant women^43^.

In terms of fish intake, almost 70% of the adolescent girls had consumed some fish in the last 24 h across all regions in both seasons. The seven-day food frequency data confirmed that tilapia was more commonly consumed in the HS and MS areas than in the LS, FW and PP areas; this finding can be explained by the abundance of tilapia in saline regions and its recent increase in commercial production^58,59^. Frequent consumption of tilapia may have played an important role in maintaining the normal concentrations of vitamin D3 among the adolescent girls.

Despite the huge potential of fisheries and aquaculture to improve the nutrition situation in Bangladesh, the number of researches conducted is very low regarding the understanding of how aquatic environment and fish consumption are associated with nutritional status of the vulnerable groups, particularly the adolescent girls. Fish is a valuable contributor to the reference nutrient intakes for a variety of micronutrients. This study was such attempt to look into the factors contributing to different micronutrient deficiencies in adolescent females in Bangladesh's west coastal areas. For aquaculture ecoregions like Bangladesh, this research is crucial, thus we measured the micronutrient status, food intake, fish intake, and nutritional status of the adolescent, through two seasonal surveys. The study improved our understanding to establish a linkage among micronutrient status, aquaculture-ecological zone and seasonality.

One of the major limitations of this study is that we were only able to measure dietary diversity rather than adequacy. Hence, no conclusions can be drawn about to what extent dietary intake of adolescent girls influenced micronutrient status. Not measuring the hemoglobin level to estimate anemia prevalence among the girls is another limitation of the study. The influences of seasonality on food security, dietary diversity and nutritional status are widely accepted. A study in rural Bangladesh showed seasonality was a key determinant of dietary diversity^60^. In addition to a distinct salinity zone effect, this study found seasonal variations in the prevalence of vitamin D insufficiency, vitamin A deficiency, and iron deficiency among adolescent girls living in coastal areas.

## Conclusions

Adolescent girls living across aquatic ecological zones in southern coastal areas of Bangladesh are seasonally at risk of multiple micronutrient insufficiencies and deficiencies. Attention should be paid to the high rates of vitamin (OH)D insufficiency, particularly in freshwater areas, and seasonal iron deficiency in the high saline zone. The results can support the development of targeted policies to improve the micronutrient status of this vulnerable group.

## Supplementary Information

Supplementary Information.

## Data Availability

Data described in the manuscript will be made available as per reasonable request to the corresponding author.

## Abbreviations

AGP: Alpha-1-acid glycoprotein
AOR: Adjusted odds ratio
BDHS: Bangladesh demographic health survey
CRP: C-reactive protein
EAR: Estimated average requirement
FW: Fresh water
HS: High saline
ID: Iron deficiency
IDA: Iron deficiency anemia
IDD: Iodine deficiency disorder
IMMANA: Innovative methods and metrics for agriculture and nutrition actions
LS: Low saline
LSHTM: London school of hygiene and tropical medicine
MD: Mean difference
MS: Medium saline
NSTU: Noakhali Science and Technology University
PCA: Principal component analysis
PP: Processing plant
PP: Percentage point
Ref: Reference
SD: Standard deviation
SES: Socioeconomic status
UIC: Urinary iodine concentration
UID: Urinary iodine deficiency
VAD: Vitamin A deficiency
