# Supplementary figures and images for: Factors affecting the micronutrient status of adolescent girls living in complex agro-aquatic ecological zones of Bangladesh
Source: Sci Rep. 2023 Apr 24;13:6631. doi: 10.1038/s41598-023-33636-8 (PMC10126111; doi:10.1038/s41598-023-33636-8)

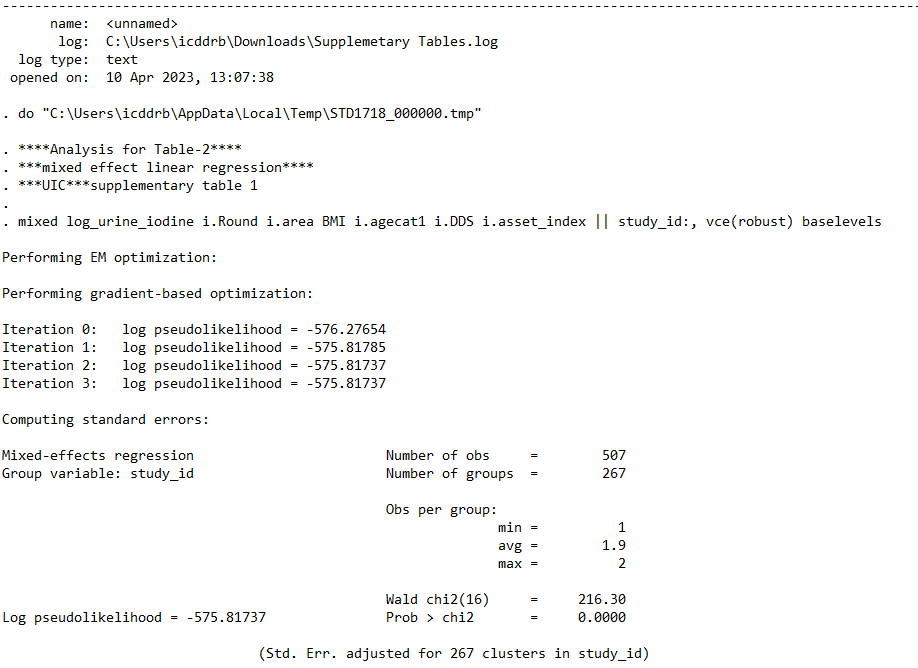


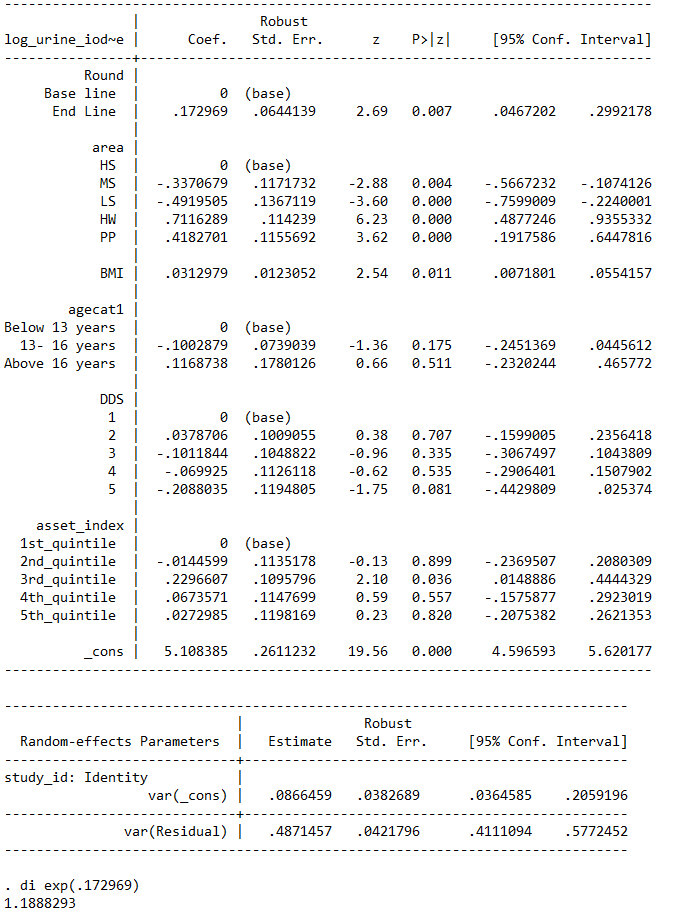


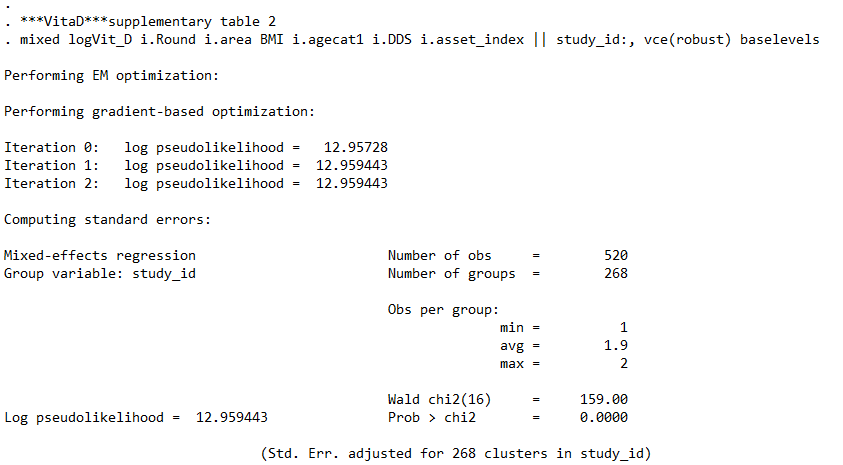


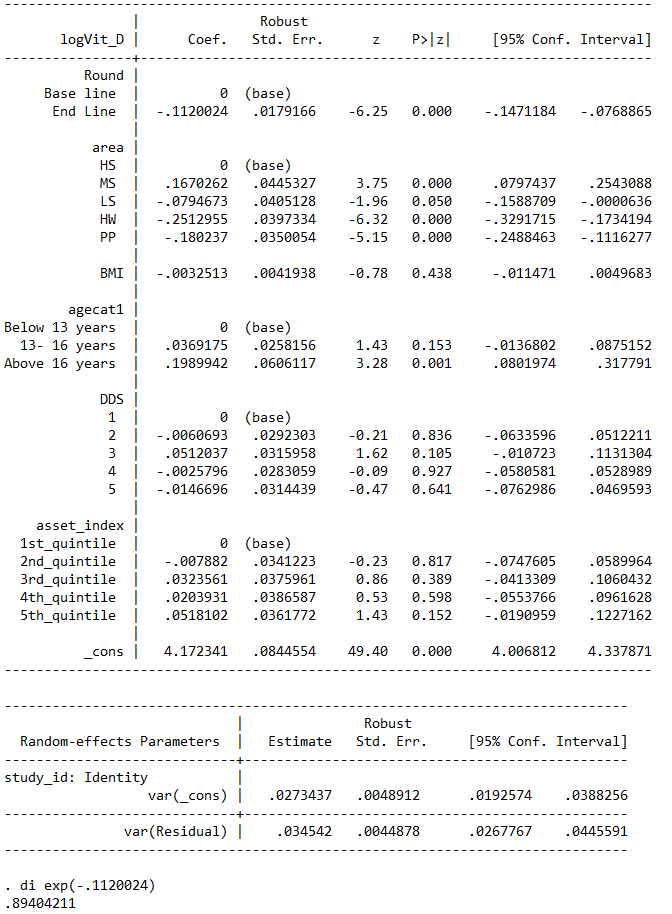


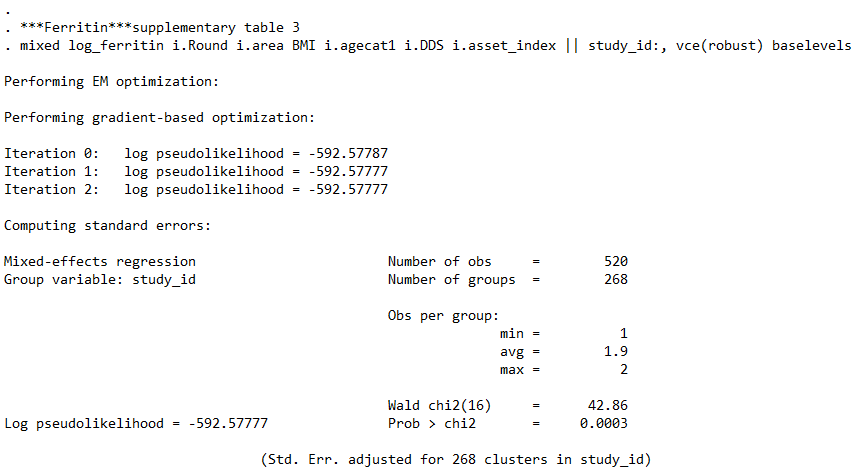


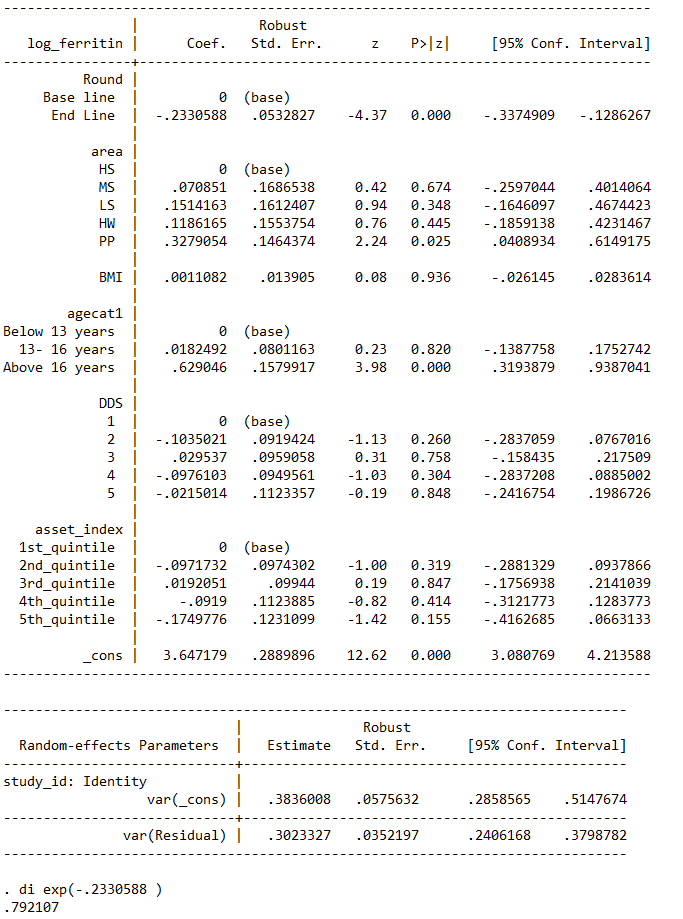


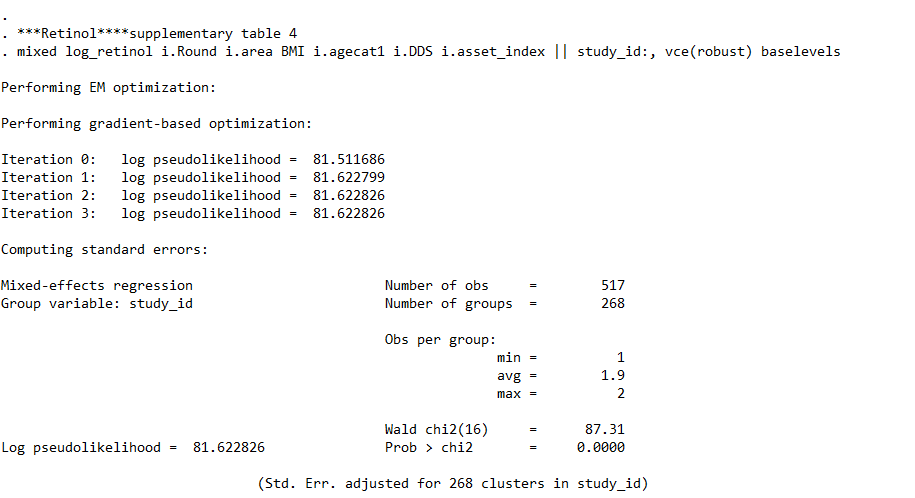


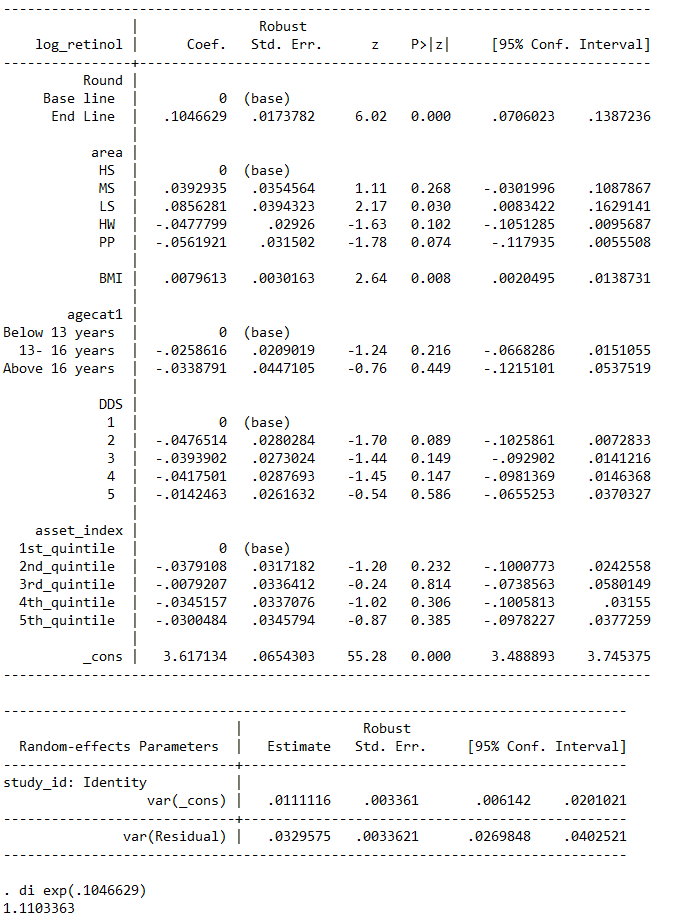


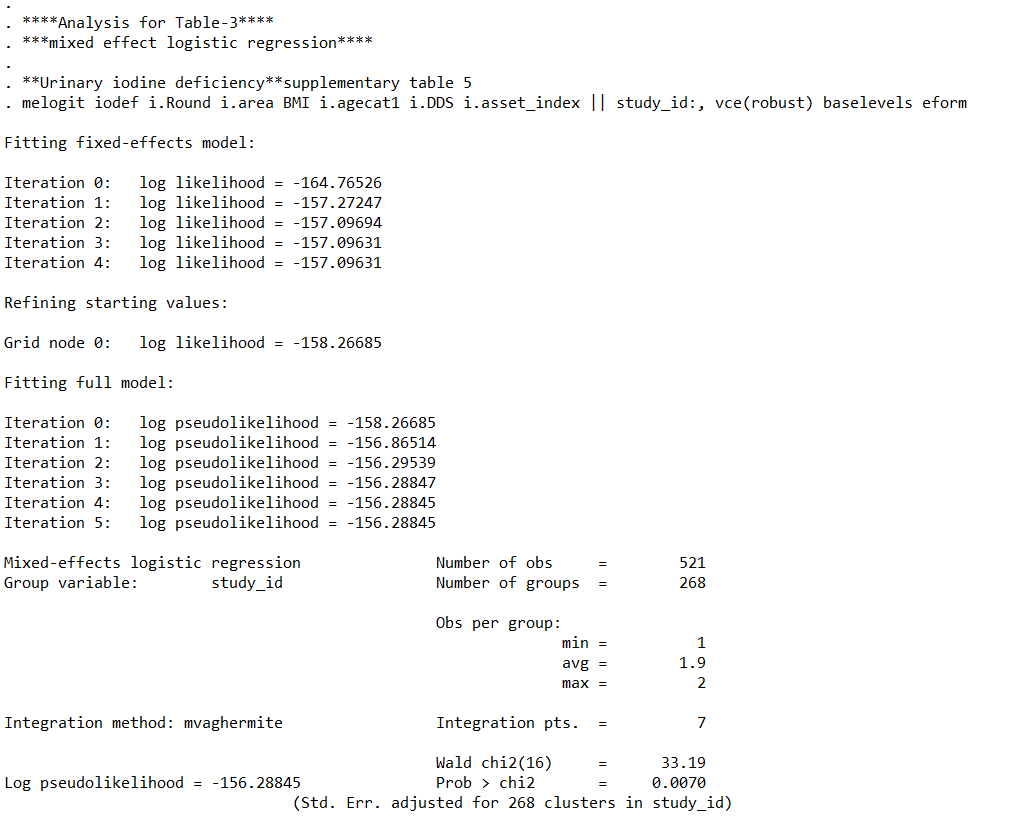


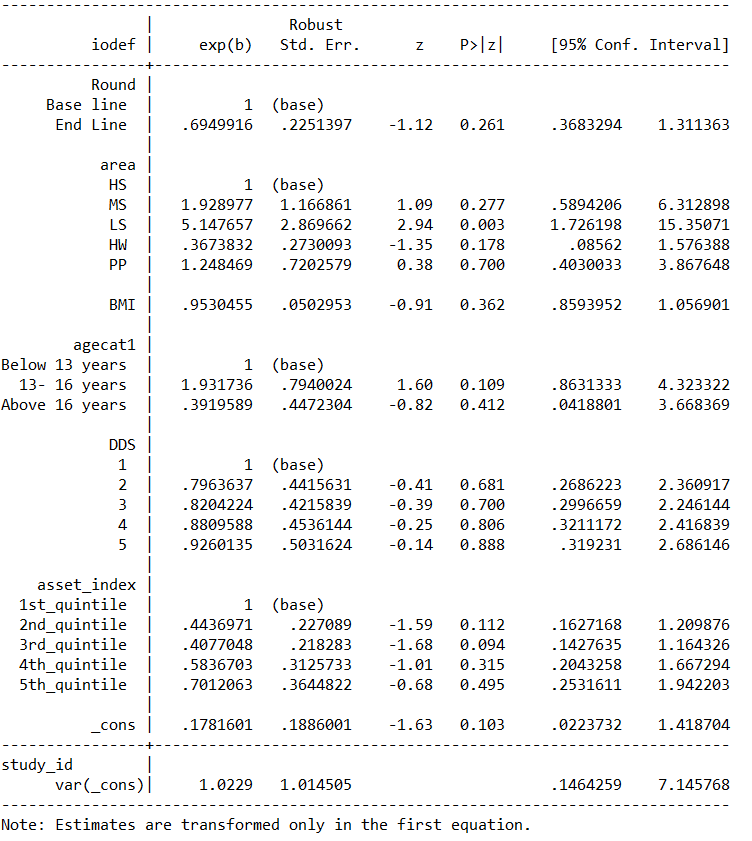


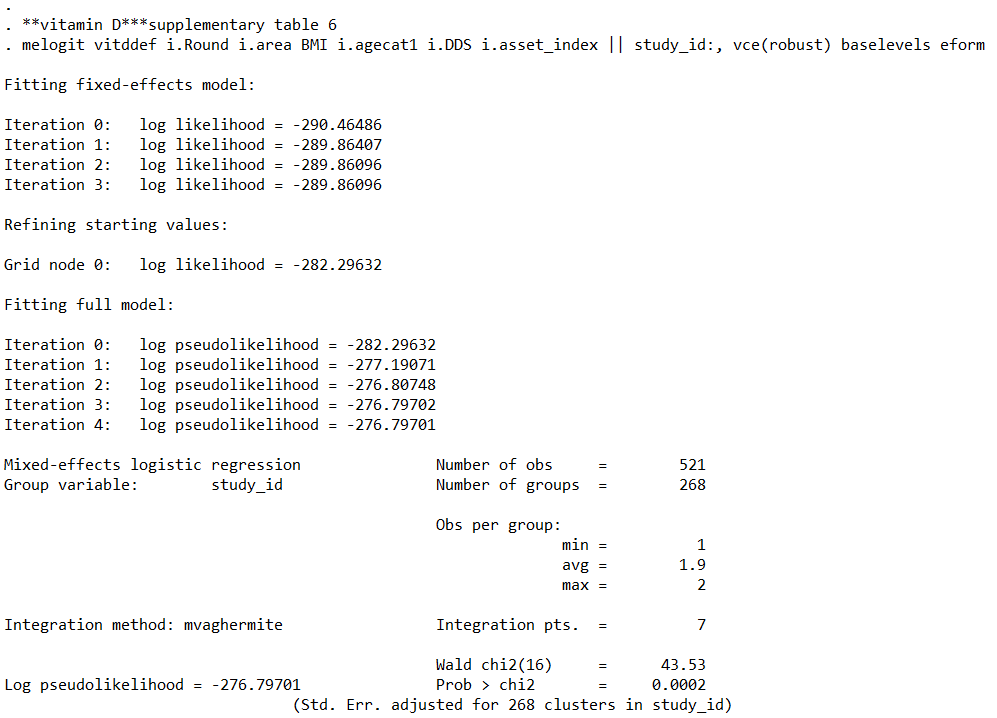


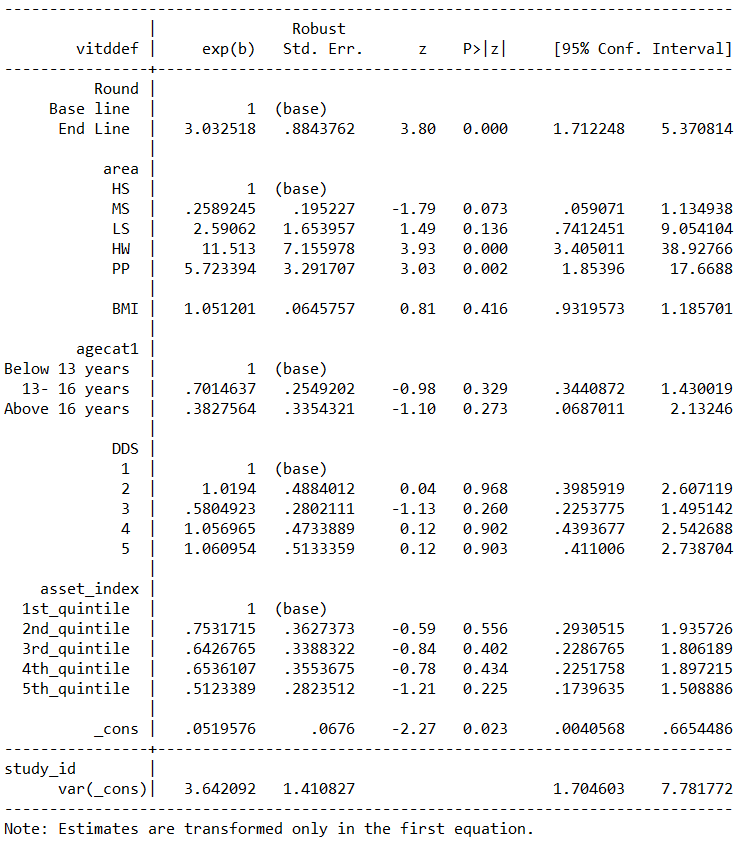


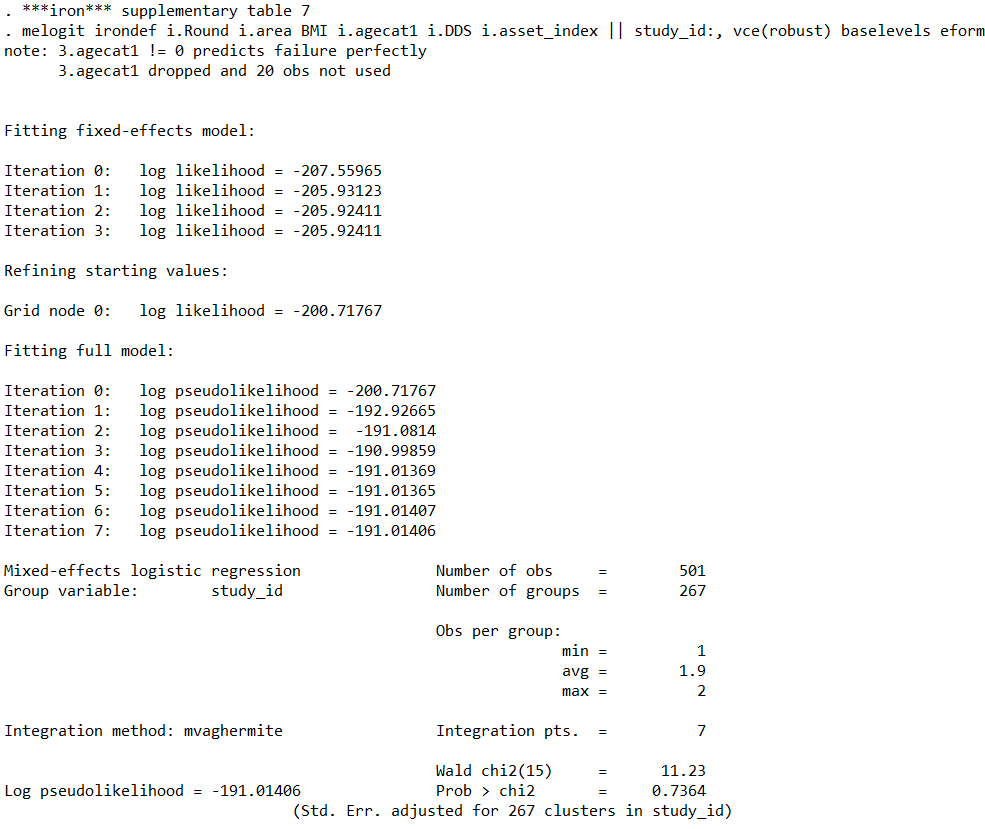


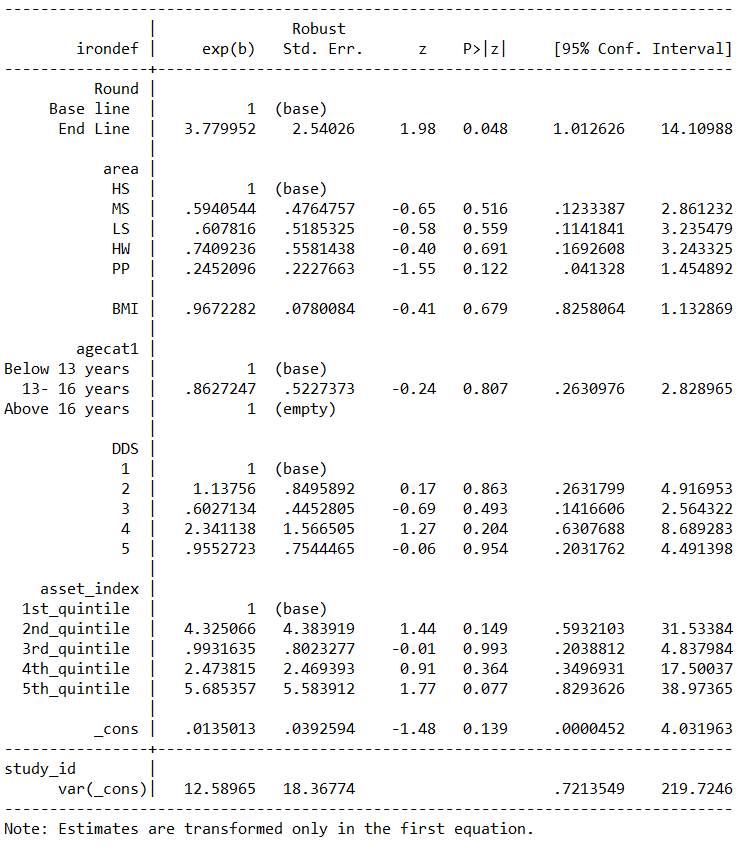


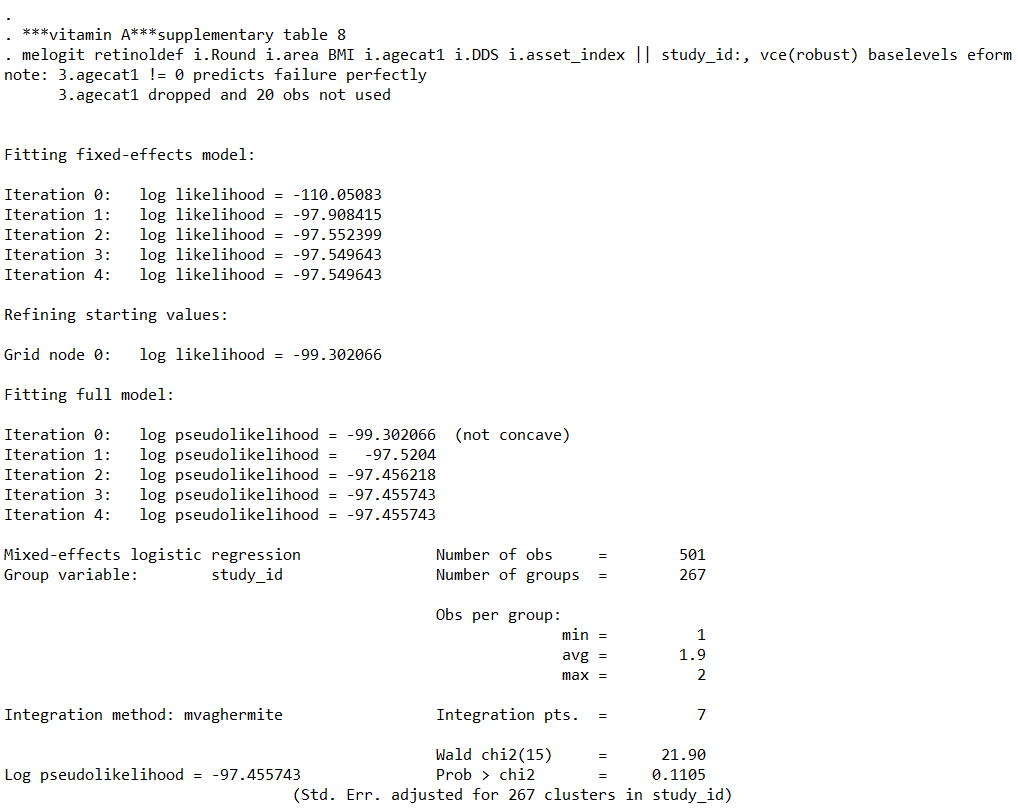


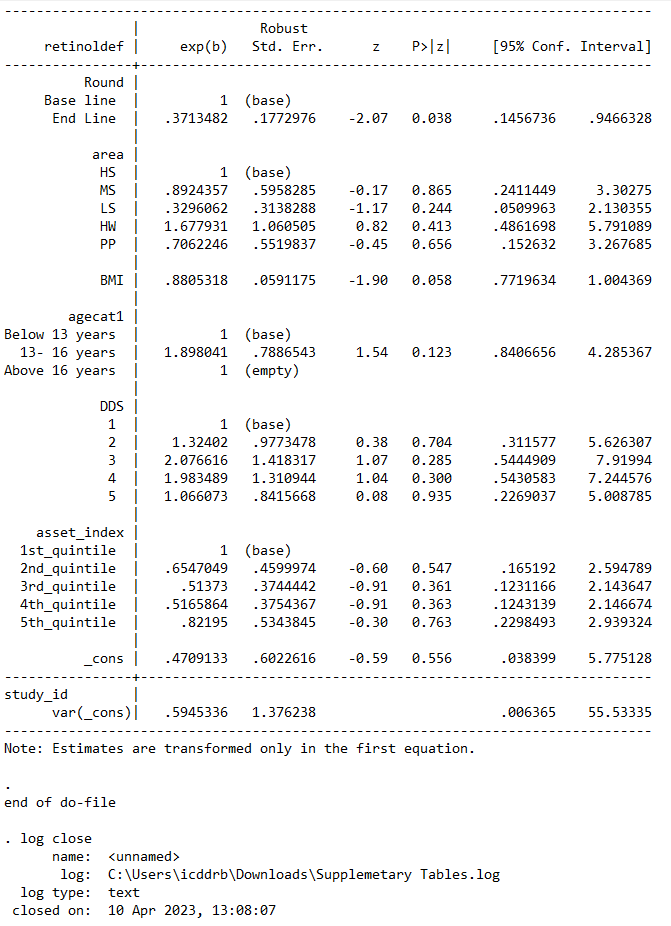

Supplement: Supplementary file 1 — Supplementary Information. [file 41598_2023_33636_MOESM1_ESM.docx]
